# Deferoxamine therapy for intracerebral hemorrhage: A systematic review

**DOI:** 10.1371/journal.pone.0193615

**Published:** 2018-03-22

**Authors:** Liling Zeng, Li Tan, Haijun Li, Qixin Zhang, Yongxian Li, Jianwen Guo

**Affiliations:** 1 Guangzhou University of Chinese Medicine, Guangzhou, Guangdong, China; 2 The 2nd Teaching Hospital of Guangzhou University of Chinese Medicine, Guangdong Provincial Hospital of Chinese Medicine, Guangdong Province Key Laboratory of Emergency Medicine of TCM, Guangzhou, Guangdong, China; Universita Politecnica delle Marche, ITALY

## Abstract

Intracerebral hemorrhage (ICH) is a significant cause of morbidity and mortality worldwide. Several recent controlled trials have reported that deferoxamine (DFX) therapy appears to be effective for ICH. The aim of this study was to perform a systematic review of DFX therapy for ICH patients and evaluate the efficacy and safety of DFX therapy for ICH patients. We searched Medline, Embase, the Cochrane Database of Systematic Reviews, clinicaltrials.gov, all Chinese databases and the reference lists of all included studies and review articles. We then performed a systematic review of studies involving the administration of DFX following ICH. Only two studies were included, a prospective, randomized clinical trial and a prospective,observational cohort study with concurrent groups. Qualitative analysis of each study revealed one randomized controlled trial of moderate quality with a moderate risk of bias and one observational cohort study of moderate quality with a moderate risk of bias. DFX may be an effective treatment for edema in patients with ICH. However, due to the small number of trials and small sample sizes of these trials, insufficient evidence exists to determine the effect of DFX on neurologic outcomes after ICH and the safety of this intervention. Further investigation is required before DFX can become a routine treatment for ICH.

## Introduction

Spontaneous intracerebral hemorrhage (sICH), a subtype of stroke, is caused by the rupture of blood vessels in the brain and is a significant cause of morbidity and mortality worldwide[1]. Approximately 40% of patients with ICH die within 30 days, and most survivors are left with severe disability[2, 3]. The acute phase of the mass effect produced by perihematomal edema (PHE) after ICH and other secondary injuries, such as neuronal death, results in neurological deterioration and poor prognosis[4]. However, no specific treatment for PHE and other secondary injuries after ICH currently exists beyond supportive and aggressive medical care[5, 6].

Faced with the limitation of current therapies, deferoxamine (DFX), a potent iron chelator, has become a promising neuro-protective drug that has been repeatedly tested in several studies. Both preliminary experimental and clinical trials has showed improved efficacy with DFX treatment for ICH[7]. Furthermore, a systematic review of DFX for experimental ICH demonstrated that DFX was effective in experimental ICH[8]. Notwithstanding these studies, it is premature to draw conclusion that DFX has sufficient efficacy and safety for patients with ICH due to the limited number of studies involving clinical trials. Furthermore, a search of the Database of Abstracts of Reviews of Effects, Cochrane Database of Systematic Reviews (CDSR), PROSPERO, Medline and Embase reveals the absence of any systematic review on this topic, except for one on animals[8]. Therefore, it is necessary to carry out an exhaustive literature review to assess all available evidence and explore current knowledge of DFX therapy in the clinical scenario of nonsurgical sICH.

## Materials and methods

The review protocol has been registered in PROSPERO, and the registration number is 42016036660.

### Research question and objectives

This review aims to answer the following research question: is DFX an effective and safe treatment for edema and hematoma in patients with ICH? The primary objective is to assess the efficacy of DFX for hematoma and edema absorption after ICH; the secondary objective is to assess whether DFX improves neurologic outcomes after ICH and whether it causes adverse events.

### Eligibility criteria for the review (PICOS elements)

Detailed information on the participants, interventions, comparisons, outcomes and types of studies (PICOS) included are provided in Table 1. Since clinical trials on this topic are rare, and no clear definition of the choice of the treatment window currently exists, the decision to initiate DFX treatment up to 24 h after ICH symptom onset is based on data from animal studies, which is currently considered the most acceptable treatment window[9].

**Table 1:** Prespecified eligibility criteria.

Participants	Interventions	Comparison	Outcomes	Study types
1) age >18years; 2) spontaneous ICH confirmed by CT; 3) onset within 24 h.	Patients in the experimental group received intravenous injections of DFX 32 mg/kg daily from the first admission day within 24 h after the onset for 3 consecutive days.	Placebo control or blank control: patients in the control group did not receive DFX.	Only studies using one or more of these tools were eligible. The primary objectives were to assess the efficacy of DFX for hematoma and edema absorption. The secondary objectives were to assess whether DFX can improve neurologic outcomes after ICH and cause adverse events.	An RCT or a controlled observational cohort study was included in the review.

ICH, intracerebral hemorrhage; CT, computed tomography; RCT, randomized controlled trial; DFX, deferoxamine.

### Study design

Scoping searches suggested a paucity of research in this area; therefore we decided to apply a high level of sensitivity and low specificity to the search process and to include not only randomized controlled trials (RCTs) but also high-quality, non-RCT studies. All available published data on DFX therapy for patients with ICH were selected for analysis, provided that all the following data were available:

1. The data source was a controlled clinical trial or a controlled observational cohort study.
2. The study design included the comparison of the efficacy or safety of DFX therapy for patients with ICH in both groups.
3. The interventions included medication use, quantity, methods of medication administration, medication dependence, and reported side effects.

### Search strategy

A comprehensive search was undertaken, using the following resources:

1. Electronic databases: CDSR, Embase (1980 to 15 Sep 2016), Ovid Medline (R) (1950 to 15 Sep 2016), clinicaltrials.gov (from inception to 15 Sep 2016),China National Knowledge Infrastructure (1950 to 15 Sep 2016), VIP Database for Chinese Technical Periodicals, Wanfang Database, and Chinese Biomedical Literature Database (1950 to 15 Sep 2016).
2. The following search terms were used: intracerebral h(a)emorrhage OR ICH OR intracranial h(a)emorrhage OR h(a)emorrhagic stroke OR stroke AND deferoxamine OR DFX OR desferrin OR Desferal OR desferrioxamine OR deferoxaminum OR deferoxamine mesylate OR desferrioxamine mesylate OR DFX OR DFM OR DFOM OR DFO OR NOT animal, combined using Boolean logic (AND, OR, NOT). To increase sensitivity, no restrictions were placed on language or study/ trial/review type.
3. Grey literature sources: three medical websites (http://www.doc88.com/daokebabakefu; http://www.medlive.cn/; http://www.dxy.cn/) were contacted for recent, or in-progress.
4. The following journals were searched by hand from Jan. 2004 to 15 Sep 2016: Stoke; Neurology; Surgical Neurology; Journal of Clinical Neurology; Stroke and Nervous Diseases; and Chinese Journal of Neurology.

### Applying selection/eligibility criteria to search results

**Fig 1:**
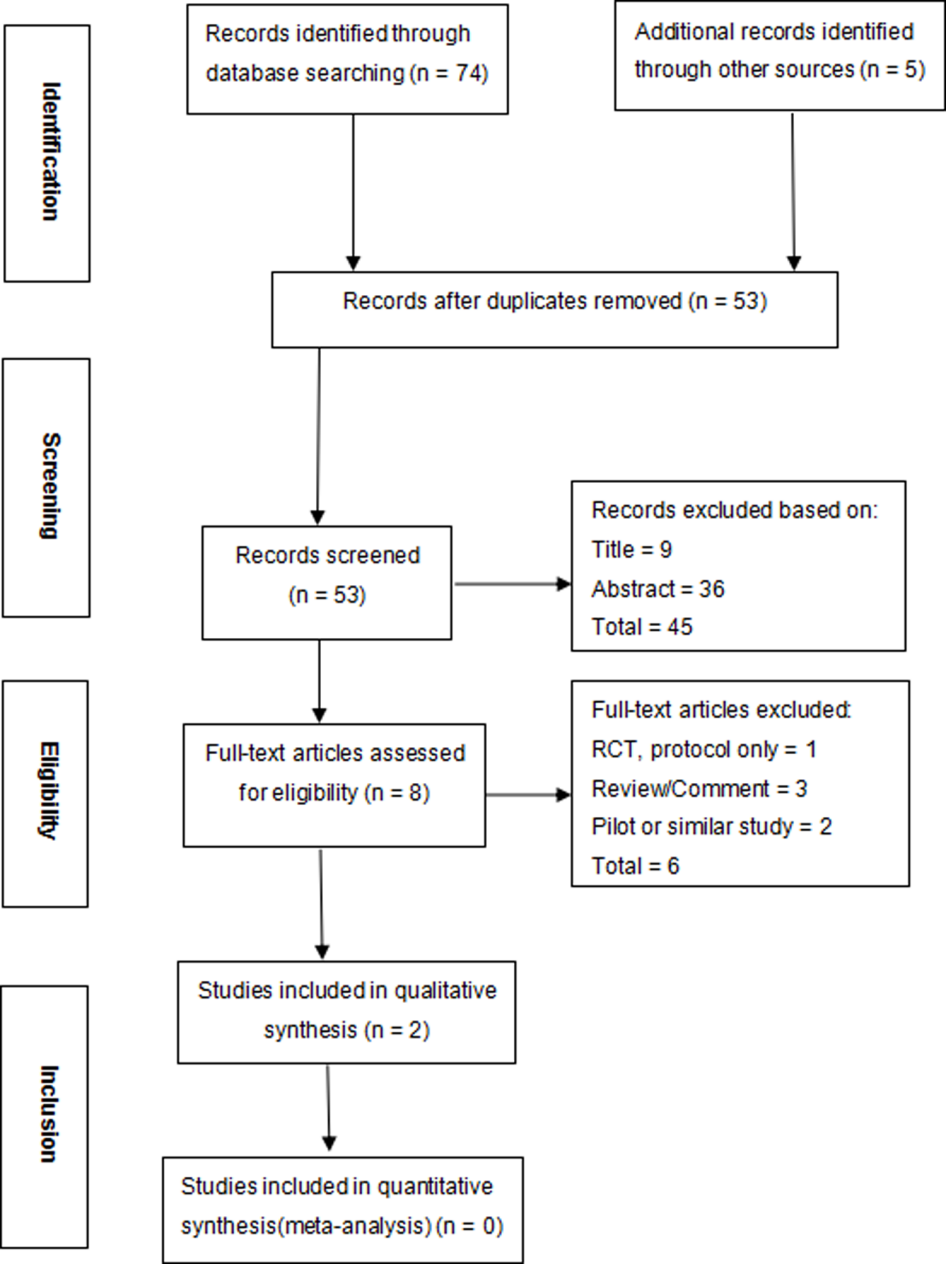
PRISMA 2009 flow diagram. PRISMA, Preferred Reporting Items for Systematic Reviews and Meta-Analyses; RCT, randomized controlled trial.

Search results were merged using Endnote Reference Manager 7 software, and duplicates/multiple reports were removed, leaving 53 articles. Forty-five reports were excluded after review of the title and abstract. A flow chart based on Preferred Reporting Items for Systematic Reviews and Meta-Analyses (PRISMA)[10] is shown in Fig 1.

Reasons for exclusion included animal experimentation, non-ICH- induced edema, and study type. The review process was independently undertaken by two reviewers (Liling Zeng and Haijun Li), with blinding to reduce bias. Disagreements were addressed by discussion with a third reviewer (Yongxian Li). The full texts of 8 studies were sought. These full-text studies were independently examined by both authors for compliance with the eligibility criteria shown in Table 2; 6 studies were excluded[7, 11–15], and two trials[16, 17], including one RCT and one prospective, controlled, observation cohort study, were deemed suitable for inclusion. The basic study design characteristics were collected and tabulated in Table 3, using a custom-designed data extraction form designed according to the Cochrane Review checklist 7.3a[18].

**Table 2:** Characteristics of the 8 studies for full-text review.

Author (year)	Title	Journal and database UI or DOI	DFX used as intervention	Focus is ICH	Study type
Yu Y etal., (2015)	The clinical effect of deferoxamine mesylate on edema after intracerebral hemorrhage.	PLoS one 10.1371/journal.pone.0122371	Yes	Yes	RCT
Yue Sun etal., (2015)	Effect of deferoxamine mesylate on occupied volume and 90 days prognosis in patients after intracerebral hemorrhage	Chin J Neuromed 10.3760/cma.j.issn.1671-8925.2015.10.010	Yes	Yes	RCT, similar to the study of Yu Y (2015)
Gang Wuetal.,(2014)	Effects of deferoxamine in patients of spontaneous intracerebral hemorrhage	Chin J Neurosurg 10.3760/cma.j.issn.1001-2346.2014.12.013	Yes	Yes	Controlled observational cohort study
Yeatts SD etal., (2013)	High dose deferoxamine in intracerebral hemorrhage (HI-DEF) trial: rationale, design, and methods	Neurocritical Care 10.1007/s12028-013-9861-y	Yes	Yes	RCT, protocol only
Chaudhary N etal.,(2013)	Iron—potential therapeutic target in hemorrhagic stroke	World Neurosurgery 10.1016/j.wneu.2012.11.048	NA	Yes	Commentary on Selim etal (2011) and precli nical animal studies
Selim M etal., (2011)	Safety and tolerability of deferoxamine mesylate in patients with acute intracerebral hemorrhage	Stroke; a journal of cerebral circulation 10.1161/STROKEAHA.111.617589	Yes	Yes	Pilot study, N = 10. Not RCT, no control group
Hua Y etal., (2008)	Deferoxamine therapy for intracerebral hemorrhage	Acta Neurochirurgica. Supplement EBSCO AN:19066072	NA	Yes	Commentary on preclinical animal studies
Selim M etal., (2009)	Deferoxamine mesylate: a new hope for intracerebral hemorrhage: from bench to clinical trials	Stroke; a journal of cerebral circulation 10.1161/STROKEAHA.108.533125	NA	Yes	General review

RCT, randomized controlled trial; ICH, intracerebral hemorrhage; CT, computed tomography; DFX, deferoxamine; NA, not applicable.

**Table 3:** Characteristics of the two included studies.

Study ID	Participants	Intervention and control interventions	Outcomes measured and reported	Results
Yao Yu (2015) Study design: RCT	Number: 42 enrolled; all included in analysis; sICH confirmed by CT; onset within 24 h;clinical status of astable condition; mean hematoma volume (ml): intervention 15.2 ± 8.8, control 12.6 ± 10.3; over age 22; unclear gender ratio; no exclusion criteria;	Experimental group: DFX. 1) administration route:intravenous injection; 2) dose: 32 mg/kg daily; 3) Manufacturer: unclear; 4) batch NO: unclear; 5) course of treatment: from the first admission day for 3 consecutive days. Notes: the infusion rate per hour did not exceed 7.5 mg/kg, and the maximum daily dose did not exceed 6000 mg. Control group: placebo, did not receive DFX.	All outcomes were defined: 1) measurement method: the primary end-point assessment (the REV measured by CT and calculated by the formula ABC/2), secondary end-point assessment (mRS); 2) follow-up and method: 30 days after admission by telephone or face-to-face interview.	The control group’s REV on the 15^th^ day was higher than that of the experimental group; the control group’s 1–8 day and 8–15 day relative hematoma absorptions were greater than those of the experimental group (*P*<0.05); the control group’s REV on the 4^th^, 8^th^, and 15^th^ day was higher than that of the experimental group (*P*<0.05); neurological scores between the two groups did not differ significantly on the 15^th^ day (or discharge) or on the 30^th^ day.
Gang Wu (2014) Study design: prospective, controlled, observational cohort study	Number: 29 enrolled; all included in analysis; sICH confirmed by CT; onset within 24 h; clinical status of a stable condition; mean hematoma volume (ml): intervention 19.4, control 15.5; over age 18; unclear gender ratio; no exclusion criteria.	Experimental group: DFX 1) administration route: intravenous injection; 2) dose: 20 mg/kg daily; 3) manufacturer: Novartis; 4) batch NO: unclear; 5) course of treatment: from the first admission day for 3 consecutive days. Notes: none. Control group: placebo, did not receive DFX.	All outcomes were defined: 1) measurement method: the primary end point (the REV measured by MRI and calculated by the following formula: V = Σ(Sn+sn+1)×h/2) secondary end-point assessment (NIHSS); 3) follow-up and method: 90 days after admission by telephone or face-to-face interview.	The control group’s REV on the first,7^th^, and 14^th^ day were higher than those of the experimental group (*P*<0.05); the neurological function in the treatment group patients on the 7th and 14th days after ICH was better than that in the control group (*P*<0.05); however, there were no significant differences on the 90th day.

RCT, randomized controlled trial; sICH, spontaneous intracerebral hemorrhage; REV, relative edema volume; CT, computed tomography; DFX, deferoxamine; ml, milliliters; mRS, modified Rankin Scale; NIHSS, National Institutes of Health Stroke Scale; MRI,magnetic resonance imaging.

### Quality assessment

Research may vary considerably in methodological rigor, and flaws in the design or conduct of a study could introduce bias, obscuring the benefit/harm of an intervention. Each study included in a review must therefore undergo a quality assessment (QA) process[19]. The Cochrane Review Handbook (5.1.0)[18] for RCTs was chosen for this review as it covers sequence generation, allocation sequence concealment, blinding, incomplete outcome data, selective outcome reporting and other sources of bias, fully assessing the quality of a study. No established QA guidelines were available on cohort studies, and the assessment instrument designed for observational studies by Liu et al[20] was adapted for this analysis. The checklist of the assessment instrument includes the following key elements [20]: (1) clear definition of the study population; (2) independent assessment of outcome parameters; (3) clear definition of outcomes and outcome assessment; (4) sufficient duration of follow-up; (5) no selective loss during follow-up; and (6) identification of important confounders and prognostic factors identified. If a study did not clearly mention one of these key points, we considered that it had not been performed. As a result, the reported characteristics were probably underestimated.

## Results

We identified 79 publications from the above-described electronic search; 26 were excluded due to duplication (n = 26). We screened 53 publications based on title and abstract and excluded 45 publications due to failure to meet the inclusion criteria (n = 45). After the full-text articles were assessed for eligibility, an additional 6 publications were excluded due to protocol only (n = 1), performance of a review only (n = 3), and pilot or similar study or duplicated publication(n = 2). Finally, this systematic review included 2 articles published before 2015 that met the inclusion criteria.

In the two eligible clinical trials[17–18]with a total of 71 patients, 40 patients were treated with DFX and compared with 31 patients who were treated without DFX. One of these studies was a prospective, randomized clinical trial[17], and the other was a prospective, observational cohort study[18] with concurrent groups. Data extraction sheets and QA forms were completed for each study and are shown in Tables 3–5. A meta-analysis was not appropriate for the current review because of the small number of eligible studies and differences in the research methods. However, a brief qualitative analysis of the evidence is presented in narrative form, supplemented by Tables 4 and 5. No significant difference was observed in the mean follow-up duration between the treatment group and the control group.

**Table 4:** Study quality for RCT.

Study ID Yao Yu (2015)	Judgment	Support for judgment
Random sequence generation	Low	Randomization was performed using statistical software SPSS 13.0 (SPSS Inc., Chicago, IL, USA).
Allocation concealment	Low	Using a random number table by one researcher who was not involved in patient recruitment.
Blinding of participants	Low	The patients were not aware of the detailed information of their treatment or the group to which they belonged; however, but no details were provided regarding how the blinding was performed.
Blinding of outcome assessment	Low	The investigator in charge of evaluating the neurological scale and the Deputy Director of Neurology in charge of studying the CT data did not know to which group a patient belonged before exposing the blind.
Incomplete outcome data	High	All outcome data were available, except adverse reactions.
Selective reporting	Low	All outcome data were reported.
Other sources of bias	Low	None.
Overall assessment	Moderate	This trial is of high quality with a moderate risk of bias.

**Table 5:** Study quality for cohort study.

Study ID Gang Wu(2014)	Judgment
Clear definition of study population?	Yes
Clear definition of outcomes and outcome assessment?	Yes
Independent assessment of outcome parameters?	Yes
Sufficient duration of follow-up?	Yes
No selective loss during follow-up?	Yes
Important confounders and prognostic factors identified?	No

### Review of objectives

#### Is DFX an effective treatment for edema and hematoma in patients with intracerebral hemorrhage?

The two included trials measured and compared the relative edema volume in both the experimental and control groups at three different times.

Yu et al[17] reported that the control group’s relative edema volume on the fourth, eighth, and fifteenth day (or discharge) was higher than that of the experimental group (*P*<0.05). However, the control group’s 1–8 day and 8–15 day relative hematoma absorption were greater than those of the experimental group (*P*<0.05), suggesting that DFX might slow hematoma absorption and inhibit edema after ICH. Similarly, Wu et al[18] found that the control group’s relative edema volumes on the first, 7th, and 14th day (or discharge) were higher than those of the experimental group. Thus, DFX may be an effective treatment for edema in patients with ICH. However, this evidence was based on one RCT of moderate quality with a moderate risk of bias and one observational cohort study of moderate quality with a moderate risk of bias—that is, level 2 evidence, which was deemed inadequate for making any recommendation to introduce this intervention based on the National Institute for Health and Clinical Excellence guidelines for evidence grading[21].

#### Dose DFX improve neurologic outcomes after ICH?

In both studies, the researchers performed neurological deficit assessments, using a variety of measurement tools. Yu et al[17] reported that neurological scores between the two groups did not differ significantly on the fifteenth day (or discharge) or on the thirtieth day. However, Wu[18] reported a significantly greater reduction in neurological scores on the 7^th^ and 14^th^ days after sICH for the experimental group (mean score 11.7 ± 4.1 and 7.4 ± 2.6) than for the control group (15.1 ± 4.9 and 11.8 ± 5.6, *P*<0.05). However, no significant differences existed on the 90th day. Because of the small number of trials and small sample sizes of these trials, this review concluded that there was no sufficient evidence that DFX could improve neurologic outcomes after ICH and that further investigation was required.

#### How is the consistency in DFX treatment protocol?

Absence of uniformity or consistency was found in the two included studies, because the dose of DFX ranged from 20 to 32 mg/kg daily, and the duration of follow-up varied from 30 days to 90 days. Furthermore, although the interventions performed in both studies were with DFX, few details were provided about the manufacturer, auxiliary material or specification of the DFX.

#### Are the details about adverse events acceptable?

Both of the studies provided serious adverse event (SAE) information related to DFX. One study reported no adverse events while patients used DFX. The other authors claimed that no SAEs were associated with DFX; however, the frequency of general adverse events was not reported. This review therefore found that no robust evidence existed regarding the safety of DFX treatment in ICH patients and that further research was needed.

## Discussion

To date, no specific treatment has proven clinically effective for ICH[5, 6]. Iron chelation has been anticipated as a suggested therapeutic treatment for ICH. Understanding how iron acts to cause secondary brain damage after ICH and the mechanisms by which the brain might be affected by iron chelation may provide a new targeted therapeutic directed toward preventing or slowing secondary injuries. Secondary injuries after ICH, such as PHE and neuronal death, are the main reasons leading to poor prognosis. Levels of non-transferrin bound iron within the brain increase substantially after ICH [22, 23]and iron-mediated free radical injury is significantly associated with secondary damage, resulting in brain edema, neuronal death and poor neurologic outcomes after ICH[24–26]. These findings indicate that the improved management of iron overload and prevention of second damage may be a promising treatment for ICH [7].

Currently, mounting evidence indicates that DFX, a ferric-iron chelator, is a candidate drug for the treatment of ICH. DFX can cross the blood-brain barrier and chelate iron ions[27], with a subsequent decrease of iron accumulation in nerve tissue[28, 29]. By this mechanism, DFX exerts various neuroprotective functions, raising anticipation of its potential for ICH treatment[7].

However, potential toxic effects of DFX such as growth failure or bone abnormalities[30, 31], hearing impairment[32], visual loss[33, 34], renal toxicity[35], cardiac disease[36] and other side effects[37, 38]have been reported in previous studies, although they were not reported in the studies included in the current review. While the mechanism leading to tissue damage resulting in side effects is not known, it may be more directly related to the high DFX peak doses administered[39, 40]. Three days of DFX infusion at 62 mg/kg/day (maximum dose not to exceed 6000 mg/day) was confirmed as safe and tolerated by ICH patients[15].

This is the first systematic review of DFX in patients with ICH. Many experimental studies have confirmed the efficacy and safety of DFX in the ICH rat model, and more clinical studies of DFX in ICH patients will be launched soon. Some anecdotal and clinical evidence suggests a potentially beneficial role for DFX in the treatment of ICH[7,14]. Indeed, a high-quality, phase II, non-randomized and non-controlled study has suggested that this intervention has merit[15]. Since DFX therapy is still a new and potential treatment for ICH, and many studies of DFX are still in the early stages, few eligible clinical trials were included in this systematic review. Even though this systematic review indicates a current lack of high-quality evidence to advocate the use of DFX as a routine treatment for ICH, it provides some significant reference on study design for future research. The lack of evidence may be due to limitations, including possible publication bias, poor study quality, and the limited number of studies. These limitations are discussed below.

### Study quality

Although the highest effect size of DFX was found in the two included studies,they were more likely to overestimate the effect sizes. Moreover, we observed that incomplete outcome data, a measurement to reduce bias, was present in one study[15], and not reported in the other study[16].

### Study design

Based on preclinical data from animal studies[8], DFX treatment was initiated in the two included studies up to 24 h after ICH symptom onset and demonstrated great efficacy with no SAEs in the early time points, which may be instructive for future studies. Regarding DFX dose and course of treatment, one study used 32 mg/kg daily, and the other used 20 mg/kg daily.In both studies, DFX was administered for 3 consecutive days from the first admission day with no marked difference in efficacy. However, clinical data regarding the effectiveness and complications of DFX and other iron chelators in human patients with acute stroke, particularly in patients with ICH, are quite limited[7]. Thus, only one phase I clinical trial in humans has been performed to explore the tolerability and safety of DFX using a dose-response relationship[15], suggesting that consecutive daily infusions of DFX after ICH were feasible, well-tolerated, and not associated with excessive SAEs or mortality, which laid the groundwork for future studies to evaluate the efficacy of DFX in ICH.

### Limitations

The limited number of studies may be the main limitation in this review, resulting in the lack of evidence for the efficacy and safety of DFX in ICH. The reasons for the limited number of high quality studies may be because DFX has attracted attention only recently, and there is still a lack of evidence-based medical research on study design, including the choice of subjects, dose of DFX and outcome measures.In addition, some clinical trials of DFX in ICH patients are still ongoing and unpublished.

## Supporting information

S1 File PRISMA 2009 checklist. (PDF)

S2 File Data set. (DOC)
