# Supplementary material for: Deferoxamine therapy for intracerebral hemorrhage: A systematic review
Source: PLoS One. 2018 Mar 22;13(3):e0193615. doi: 10.1371/journal.pone.0193615 (PMC5863956; doi:10.1371/journal.pone.0193615)
Supplement: S2 File — (DOC) [file pone.0193615.s002.doc]

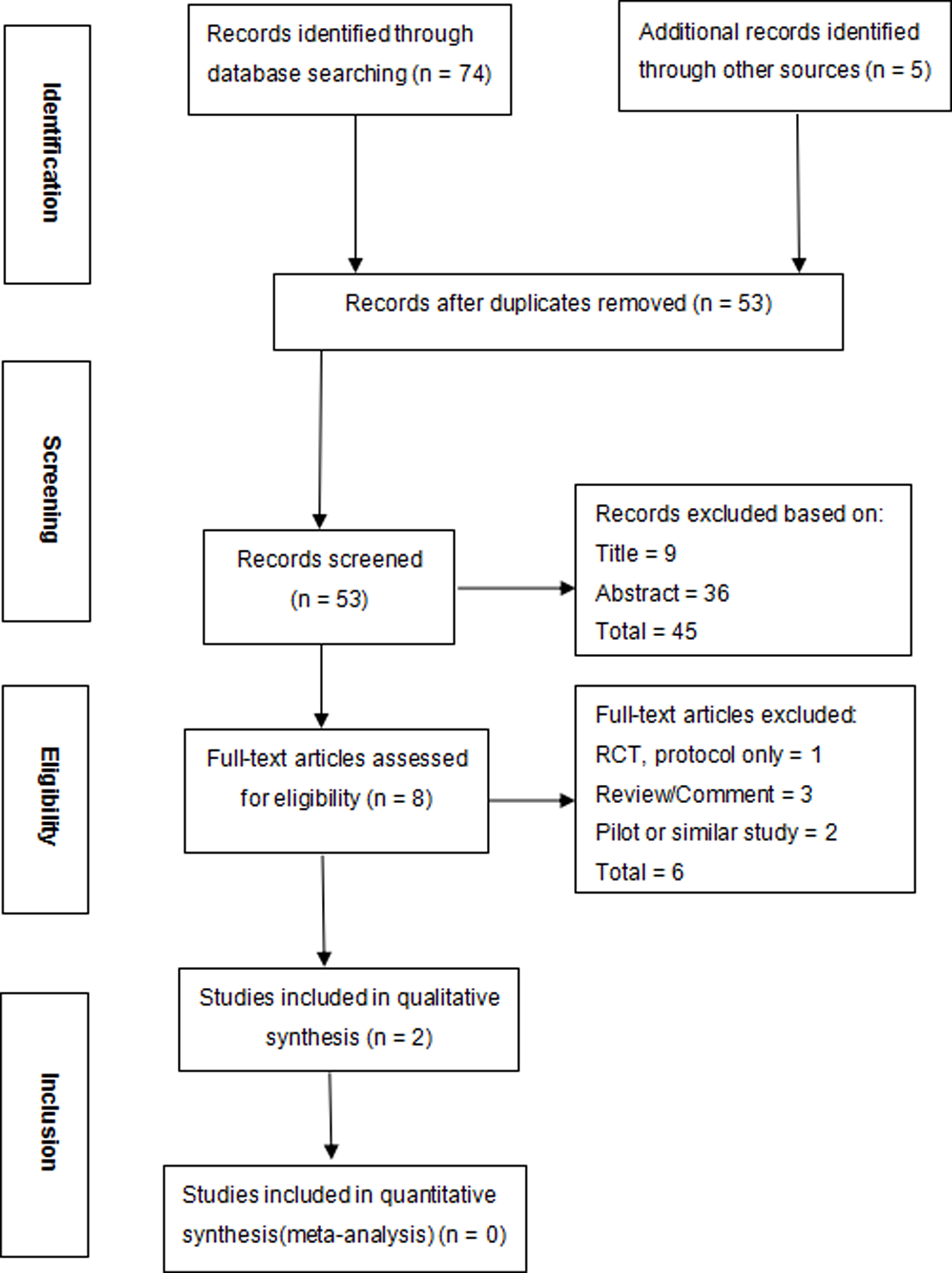


**Fig 1 PRISMA 2009 flow diagram**. PRISMA, Preferred Reporting Items for Systematic Reviews and Meta-Analyses; RCT, randomized controlled trial.

**Table 1. Prespecified eligibility criteria.**

| **Participants** | **Interventions** | **Comparison** | **Outcomes** | **Study types** |
| --- | --- | --- | --- | --- |
| 1. age >18years; 2) spontaneous ICH confirmed by CT; 3) onset within 24 h. | Patients in the experimental group received intravenous injections of DFX 32 mg/kg daily from the first admission day within 24 h after the onset for 3 consecutive days. | Placebo control or blank control: patients in the control group did not receive DFX. | Only studies using one or more of these tools were eligible. The primary objectives were to assess the efficacy of DFX for hematoma and edema absorption. The secondary objectives were to assess whether DFX can improve neurologic outcomes after ICH and cause adverse events. | An RCT or a controlled observational cohort study was included in the review. |

*ICH, intracerebral hemorrhage; CT, computed tomography; RCT, randomized controlled trial; DFX, deferoxamine.

**Table 2 Characteristics of the 8 studies for full-text review**

| **Author (year)** | **Title** | **Journal and database UI or DOI** | **DFX used as intervention** | **Focus is ICH** | **Study type** |
| --- | --- | --- | --- | --- | --- |
| Yu Y etal., (2015) | The clinical effect of deferoxamine mesylate on edema after intracerebral hemorrhage. | PLoS one  10.1371/journal.pone.0122371 | Yes | Yes | RCT |
| Yue Sun etal., (2015) | Effect of deferoxamine mesylate on occupied volume and 90 days prognosis in patients after intracerebral hemorrhage | Chin J Neuromed  10.3760/cma.j.issn.1671-8925.2015.10.010 | Yes | Yes | RCT, similar to the study of Yu Y (2015) |
| Gang Wuetal.,(2014) | Effects of deferoxamine in patients of spontaneous intracerebral hemorrhage | Chin J Neurosurg  10.3760/cma.j.issn.1001-2346.2014.12.013 | Yes | Yes | Controlled observational cohort study |
| Yeatts SD etal., (2013) | High dose deferoxamine in intracerebral hemorrhage (HI-DEF) trial: rationale, design, and methods | Neurocritical Care  10.1007/s12028-013-9861-y | Yes | Yes | RCT, protocol only |
| Chaudhary N etal.,(2013) | Iron--potential therapeutic target in hemorrhagic stroke | World Neurosurgery  10.1016/j.wneu.2012.11.048 | NA | Yes | Commentary on Selim etal (2011) and precli nical animal studies |
| Selim M etal., (2011) | Safety and tolerability of deferoxamine mesylate in patients with acute intracerebral hemorrhage | Stroke; a journal of cerebral circulation  10.1161/STROKEAHA.111.617589 | Yes | Yes | Pilot study, N=10. Not RCT, no control group |
| Hua Y etal., (2008) | Deferoxamine therapy for intracerebral hemorrhage | Acta Neurochirurgica. Supplement  EBSCO AN:19066072 | NA | Yes | Commentary on preclinical animal studies |
| Selim M etal., (2009) | Deferoxamine mesylate: a new hope for intracerebral hemorrhage: from bench to clinical trials | Stroke; a journal of cerebral circulation  10.1161/STROKEAHA.108.533125 | NA | Yes | General review |

RCT, randomized controlled trial; ICH, intracerebral hemorrhage; CT, computed tomography; DFX, deferoxamine; NA, not applicable.

**Table 3 Characteristics of the two included studies**

| **Study ID** | **Participants** | **Intervention and control interventions** | **Outcomes measured and reported** | **Results** |
| --- | --- | --- | --- | --- |
| Yao Yu (2015)  Study design: RCT | Number: 42 enrolled; all included in analysis; sICH confirmed by CT; onset within 24 h;clinical status of astable condition; mean hematoma volume (ml): intervention 15.2 ± 8.8, control 12.6 ± 10.3; over age 22; unclear gender ratio; no exclusion criteria; | Experimental group: DFX. 1) administration route:intravenous injection; 2) dose: 32 mg/kg daily; 3) Manufacturer: unclear; 4) batch NO: unclear; 5) course of treatment: from the first admission day for 3 consecutive days. Notes: the infusion rate per hour did not exceed 7.5 mg/kg, and the maximum daily dose did not exceed 6000 mg. Control group: placebo, did not receive DFX. | All outcomes were defined: 1) measurement method: the primary end-point assessment (the REV measured by CT and calculated by the formula ABC/2), secondary end-point assessment (mRS); 2) follow-up and method: 30 days after admission by telephone or face-to-face interview. | The control group’s REV on the 15^th^ day was higher than that of the experimental group; the control group’s 1 – 8 day and 8 –15 day relative hematoma absorptions were greater than those of the experimental group (*P*<0.05); the control group’s REV on the 4^th^ , 8^th^, and 15^th^ day was higher than that of the experimental group (*P*<0.05); neurological scores between the two groups did not differ significantly on the 15^th^ day (or discharge) or on the 30^th^ day. |
| Gang Wu (2014)  Study design: prospective, controlled, observational cohort study | Number: 29 enrolled; all included in analysis; sICH confirmed by CT; onset within 24 h; clinical status of a stable condition; mean hematoma volume (ml): intervention 19.4, control 15.5; over age 18; unclear gender ratio; no exclusion criteria. | Experimental group: DFX 1) administration route: intravenous injection; 2) dose: 20 mg/kg daily; 3) manufacturer: Novartis; 4) batch NO: unclear; 5) course of treatment: from the first admission day for 3 consecutive days. Notes: none. Control group: placebo, did not receive DFX. | All outcomes were defined: 1) measurement method: the primary end point (the REV measured by MRI and calculated by the following formula: V=Σ(Sn+sn+1)×h／2) secondary end-point assessment ( NIHSS); 3) follow-up and method: 90 days after admission by telephone or face-to-face interview. | The control group’s REV on the first,7^th^, and 14^th^ day were higher than those of the experimental group ( *P*<0.05); the neurological function in the treatment group patients on the 7th and 14th days after ICH was better than that in the control group ( *P*<0.05); however, there were no significant differences on the 90th day. |

RCT, randomized controlled trial; sICH, spontaneous intracerebral hemorrhage; REV, relative edema volume; CT, computed tomography; DFX, deferoxamine; ml, milliliters; mRS, modiﬁed Rankin Scale; NIHSS, National Institutes of Health Stroke Scale; MRI,magnetic resonance imaging.

**Table 4 Study quality for RCT**

| **Study ID Yao Yu (2015)** | **Judgment** | **Support for judgment** |
| --- | --- | --- |
| Random sequence generation | Low | Randomization was performed using statistical software SPSS 13.0 (SPSS Inc., Chicago, IL, USA). |
| Allocation concealment | Low | Using a random number table by one researcher who was not involved in patient recruitment. |
| Blinding of participants | Low | The patients were not aware of the detailed information of their treatment or the group to which they belonged; however, but no details were provided regarding how the blinding was performed. |
| Blinding of outcome assessment | Low | The investigator in charge of evaluating the neurological scale and the Deputy Director of Neurology in charge of studying the CT data did not know to which group a patient belonged before exposing the blind. |
| Incomplete outcome data | High | All outcome data were available, except adverse reactions. |
| Selective reporting | Low | All outcome data were reported. |
| Other sources of bias | Low | None. |
| Overall assessment | Moderate | This trial is of high quality with a moderate risk of bias. |

**Table 5 Study quality for cohort study**

| **Study ID Gang Wu(2014)** | **Judgment** |
| --- | --- |
| Clear definition of study population? | Yes |
| Clear definition of outcomes and outcome assessment? | Yes |
| Independent assessment of outcome parameters? | Yes |
| Sufficient duration of follow-up? | Yes |
| No selective loss during follow-up? | Yes |
| Important confounders and prognostic factors identified? | No |
